# Klein tunneling near the Dirac points in metal-dielectric multilayer metamaterials

**DOI:** 10.1038/s41598-017-09899-3

**Published:** 2017-08-29

**Authors:** Lei Sun, Jie Gao, Xiaodong Yang

**Affiliations:** Department of Mechanical and Aerospace Engineering, Missouri University of Science and Technology, Rolla, Missouri 65409 USA

## Abstract

The Klein tunneling of optical waves near the Dirac points in the metal-dielectric multilayer metamaterials is theoretically investigated and demonstrated through the coupled-mode theory under the tight-binding approximation and the rigorous band structure analysis based on the transfer-matrix method. The optical analogue of Klein tunneling for the relativistic electrons passing across a potential barrier is revealed by the iso-frequency contour analysis and numerical simulation to describe the optical beam propagation and refraction across the interface of two metal-dielectric multilayer metamaterial stacks. The transmission and reflection spectra of the Klein tunneling of optical waves are also explained by the coupled mode theory.

## Introduction

Klein tunneling represents the phenomenon that the relativistic fermions can pass across a high potential barrier without the exponential damping, which is a particular property that arising from the existence of negative-energy solutions of the Dirac equation^1–5^. Although, the Klein tunneling for charge carriers acting as massless Dirac fermions in graphene heterojunctions is demonstrated^6–8^, the Klein tunneling for relativistic electrons is very difficult to be directly observed in experiments due to the required experimental condition of extremely high energy. Since Klein tunneling can be described as a wave phenomenon, optical analogues of Klein tunneling for light beam propagation in waveguide lattices and photonic crystals with conical dispersion are studied in numerical simulations^9–12^. Recently, metal-dielectric multilayer metamaterials with unique dispersion relation are utilized for realizing many intriguing optical phenomena such as negative refraction^13, 14^, sub-wavelength focusing^15, 16^, diffraction-free propagation^17–19^, and anomalous indefinite cavities^20^. The strong spatial dispersion in metal-dielectric multilayer metamaterials will induce the optical nonlocality, which is connected to the coherent coupling of surface plasmon polariton (SPP) eigenmodes propagating along the interfaces of metal and dielectric layers^21, 22^. It is shown that the degeneracy of the symmetric and anti-symmetric SPP modes in metal-dielectric multilayers forms the Dirac point at the centre of the Brillouin zone, where an effectively zero “optical mass” is realized^23, 24^. The existence of the Dirac points in metal-dielectric multilayer metamaterials not only enable important research topics about the epsilon-near-zero metamaterials^23, 24^ and the nonlocal effective medium theory^25–29^, but also open new opportunities in connecting the classical electrodynamics system and the relativistic quantum mechanics system^30, 31^ for exploring relativistic phenomena for massless Dirac fermions in condensed matter physics such as *Zitterbewegung* effect^32, 33^, etc. Furthermore, it is worth mentioning that except for the metal-dielectric multilayer metamaterials, the Dirac point can also be found in a simple lattice of graphene sheets embedded in a dielectric host medium, which can raise the topological phase transition in the system^34^.

In this work, the Klein tunneling of optical waves near the Dirac points in the lossless metal-dielectric multilayer metamaterials is investigated and demonstrated through theoretical analysis and numerical simulation. Compared with the binary dielectric superlattices that previously applied to study the Klein tunneling for optical waves^9, 10^, the lossless metal-dielectric multilayer metamaterials not only have a simpler structure, which can be tuned to achieve the Klein tunneling easily, but also possess a special band structure associated with the two SPP eigenmodes, which can be degenerated to form the Dirac point, resulting in a better analogue to the Klein tunneling in graphene. According to the specified coupled-mode theory under the tight-binding approximation, the connection between the metal-dielectric multilayer metamaterials and the relativistic fermions system described by the Dirac equation is revealed. The band structures of metal-dielectric multilayer metamaterials are analyzed based on the transfer-matrix method to reveal both the frequency regions of Klein tunneling around the Dirac points and the Klein tunneling zone in the wave vector space. The optical analogue of Klein tunneling for the relativistic electrons passing across a potential barrier is further illustrated by the iso-frequency contour analysis to describe the optical wave propagation and refraction across the interface between two metal-dielectric multilayer metamaterial stacks, which is coincident with the optical beam tunneling visualized from numerical simulation. Furthermore, the transmission and reflection spectra of the Klein tunneling of optical waves across the interface of two multilayer metamaterial stacks are also explained based on the coupled-mode theory.

## Results

### Coupled-mode theory and Dirac equation

**Figure 1:**
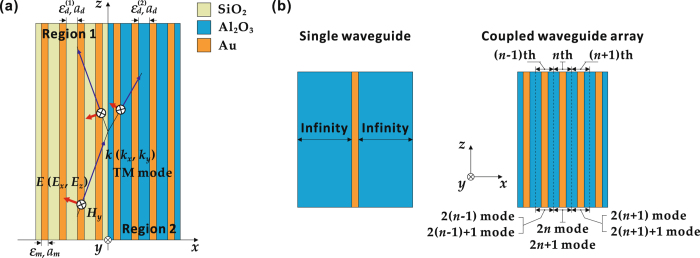
(**a**) Schematic of two metal-dielectric multilayer metamaterial stacks with an interface with respect to the TM polarized light, consisting of the Au-SiO_2_ multilayer stack in Region 1 and the Au-Al_2_O_3_ multilayer stack in Region 2. (**b**) Schematics of a single dielectric-metal-dielectric waveguide and the coupled waveguide array with two eigenmodes marked as the 2*n* mode and the 2*n* + 1 mode for the *n*th waveguide.

As displayed in Fig. 1(a), two metal-dielectric multilayer metamaterial stacks with an interface located at *x* = 0 are considered. The multilayer metamaterial stack along the negative *x*-direction in Region 1 is composited of infinite alternating layers of gold (Au) and fused silica (SiO_2_), while the multilayer metamaterial stack along the positive *x*-direction in Region 2 is made of infinite alternating layers of gold (Au) and alumina (Al_2_O_3_). The light propagating inside the metamaterials in the *x*-*z* plane is TM (transverse magnetic) polarized. The thickness of the Au layer is *a*_*m*_ = 10 nm, while the thicknesses of the SiO_2_ layer and the Al_2_O_3_ layer are the same as *a*_*d*_ = 400 nm. The permittivity of Au is describe by the Drude model as ${\varepsilon }_{m}={\varepsilon }_{\infty }-{\omega }_{p}^{2}/\omega \,(\omega +{\bf{i}}\gamma )$ with the offset constant *ε*_∞_ = 9 and the plasma frequency *ω*_*p*_ = 13.8 × 10^12^ rad/s^35^. The damping factor *γ* in the Drude model is set to be zero in the current analysis in order to emphasize the SPP eigenmodes that associated with the Klein tunneling. In addition, the permittivities of SiO_2_ and Al_2_O_3_ are simplify set as ${\varepsilon }_{d}^{\mathrm{(1)}}=1.45\times 1.45$ ^36^ and ${\varepsilon }_{d}^{\mathrm{(2)}}=1.76\times 1.76$ ^37^, respectively. Note that in order to distinguish the same physical quantities in either the Au-SiO_2_ multilayer or the Au-Al_2_O_3_ multilayer, the superscripts of (1) and (2) are applied.

As depicted in Fig. 1(b), each metal-dielectric multilayer stack can be regarded as the combination of infinite coupled dielectric-metal-dielectric waveguide array, where a single waveguide with two eigenmodes can be numbered by the index of *n*, while the two eigenmodes supported in the *n*th waveguide are numbered as the 2*n* mode and the 2*n* + 1 mode, respectively^38^. Therefore, the band structure of each metal-dielectric multilayer stack can be described by the coupled-mode theory^39^. Under the tight-binding approximation, the coupled-mode theory for each of the two metal-dielectric multilayer stacks can be expressed as

1 $$-\,({\bf{i}}{\rm{d}}/{\rm{d}}z+{\beta }_{0}-\delta ){A}_{2n}=-\kappa {A}_{2n-1}+\kappa {A}_{2n+1}$$

2 $$-\,({\bf{i}}{\rm{d}}/{\rm{d}}z+{\beta }_{0}+\delta ){A}_{2n+1}=\kappa {A}_{2n}-\kappa {A}_{2n+2}$$

where *β*_0_ presents the propagation constant of optical wave, *δ* presents the propagation constant difference of mode, and *κ* presents the mode coupling strength [Appendix 1]. Moreover, the wave functions *A*_2*n*_ and *A*_2*n*+1_ individually denote the 2*n* mode and the 2*n* + 1 mode in the *n* th waveguide, while the wave functions *A*_2*n*−1_ and *A*_2*n*+2_ individually denote the 2(*n* − 1) + 1 mode in the (*n* − 1)th waveguide and the 2(*n* + 1) mode in the (*n* + 1)th waveguide. According to the Bloch’s theorem, Eqs (1) and (2) can be solved by setting the wave functions *A*_2*n*−1_, *A*_2*n*_, *A*_2*n*+1_, and *A*_2*n*+2_ as

3 $$\{\begin{array}{rcl}{A}_{2n-1} & = & B\,\exp ({\bf{i}}\beta z+{\bf{i}}(n-\mathrm{1)}\phi )\\ {A}_{2n} & = & A\,\exp ({\bf{i}}\beta z+{\bf{i}}n\phi )\\ {A}_{2n+1} & = & B\,\exp ({\bf{i}}\beta z+{\bf{i}}n\phi )\\ {A}_{2n+2} & = & A\,\exp ({\bf{i}}\beta z+{\bf{i}}(n+\mathrm{1)}\phi )\end{array}$$

with the periodic phase shift *ϕ* = *k*_*x*_(*a*_*m*_ + *a*_*d*_) and the amplitudes *A* and *B* respectively for the two modes in a single waveguide. With respect to the wave functions in Eq. (3), the band structure of the metal-dielectric multilayer stacks can be obtained from Eqs (1) and (2) [Appendix 1] as

4 $${\beta }_{\pm }={\beta }_{0}\pm \sqrt{{\delta }^{2}+2{\kappa }^{2}-2{\kappa }^{2}\,\cos \,\phi }$$

In general, Eq. (4) indicates that the band structure possesses both the *β*_+_-branch and the *β*_−_-branch with respect to the frequency *ω*. The relation between the propagation constants *β*_±_ and *k*_*x*_ at a certain frequency *ω* based on Eq. (4) represents the iso-frequency contour (IFC). Since the tight-binding approximation only considers the coupling among the adjacent waveguides, the band structure predicted in Eq. (4) is only accurate around the Brillouin zone centre compared with the rigorous results based on the transfer-matrix method (see the following analysis). Nevertheless, Eqs (1) and (2) can still reveal the in-depth mechanism about the propagation of the TM polarized light in the metal-dielectric multilayer stacks. By introducing the relations of

5 $$\{\begin{array}{rcl}{A}_{2n} & = & -\,{\bf{i}}{\psi }_{1}(n,z)\\ {A}_{2n+1} & = & {\psi }_{2}(n,z)\end{array}$$

and

6 $${\psi }_{\mathrm{1,2}}(n\pm \mathrm{1,}\,z)={\psi }_{\mathrm{1,\; 2}}(\xi ,z)\pm \partial {\psi }_{\mathrm{1,2}}(\xi ,z)/\partial \xi $$

with the continuous transverse coordinate $\xi \leftrightarrow n={\rm{floor}}(x/a)=\lfloor x/a\rfloor$ under the assumption of the slow mode amplitude variation between the adjacent waveguides, where *a* = *a*_*m*_ + *a*_*d*_ is the thickness of multilayer unit cell, Eqs (1) and (2) can be rewritten as

7 $${\bf{i}}\frac{\partial }{\partial z}[\begin{array}{c}{\psi }_{1}\\ {\psi }_{2}\end{array}]=-{\bf{i}}\kappa {\sigma }_{1}\frac{\partial }{\partial \xi }[\begin{array}{c}{\psi }_{1}\\ {\psi }_{2}\end{array}]+\delta {\sigma }_{3}[\begin{array}{c}{\psi }_{1}\\ {\psi }_{2}\end{array}]-{\beta }_{0}[\begin{array}{c}{\psi }_{1}\\ {\psi }_{2}\end{array}]$$

in which $${\sigma }_{1}=[\begin{array}{cc}0 & 1\\ 1 & 0\end{array}]$$ and $${\sigma }_{3}=[\begin{array}{cc}1 & 0\\ 0 & -1\end{array}]$$ are the Pauli matrices. By comparing with the one-dimensional Dirac equation along the *x*-direction

8 $${\bf{i}}\hslash \frac{\partial \psi }{\partial t}=-{\bf{i}}c\hslash {\sigma }_{1}\frac{\partial \psi }{\partial x}+m{c}^{2}{\sigma }_{3}\psi +V(x)\psi $$

it is shown that the coupled-mode theory in Eq. (7) has the same format as the Dirac equation with the coefficient mapping relations of *κ* ↔ *c*, *δ* ↔ *mc* ^2^/*ħ*, and −*β*_0_ ↔ *V*(*x*)/*ħ*, and the variable mapping relations of *z* ↔ *t* and *ξ* ↔ *x*. Furthermore, the positive- and negative-energy solutions of the Dirac equation can be mapped into the *β*_+_- and *β*_−_-branches of the band structures in the metal-dielectric multilayer stack, respectively. Therefore, the Klein tunneling for the relativistic electrons passing across a potential barrier in the relativistic quantum mechanics can be mimicked by its optical analogue where the TM polarized light waves propagate and refract across the interface of two metal-dielectric multilayer metamaterial stacks.

### Band structure analysis for Klein tunneling

**Figure 2:**
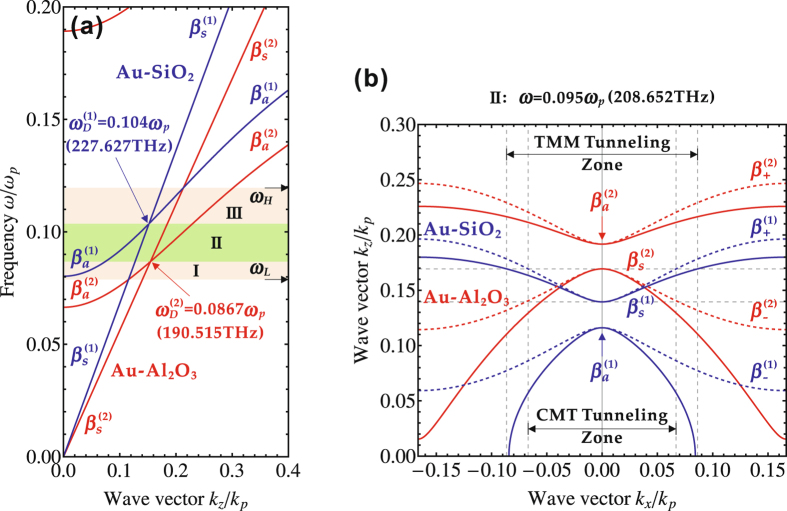
(**a**) The dispersion relations for the Au-SiO_2_ multilayer stack (blue curves) and the Au-Al_2_O_3_ multilayer stack (red curves). The symmetric mode *β*_*s*_ and the anti-symmetric mode *β*_*a*_ are marked, together the Dirac points (*ω*_*D*_) for two multilayer stacks with the superscripts of (1) and (2). The frequency band *ω* ∈ [*ω*_*L*_, *ω*_*H*_] for the Klein tunneling of optical waves is depicted as three ranges as I, II, and III. (**b**) The IFCs at the frequency *ω* = 0.095*ω*_*p*_ (208.652 THz) in the frequency range II for the Au-SiO_2_ multilayer stack (blue curves) and the Au-Al_2_O_3_ multilayer stack (red curves) according to the transfer-matrix method (solid curves) and the coupled-mode theory (dashed curves). The Klein tunneling zones in wave vector space based on the transfer-matrix method (TMM tunneling zone) and the coupled-mode theory (CMT tunneling zone) are also indicated.

In order to precisely predict the Klein tunneling of optical waves across the interface of two metal-dielectric multilayer metamaterial stacks, the rigorous band structure based on the transfer-matrix method is considered to describe the propagation of the TM polarized light in each metal-dielectric multilayer stack as

9 $$\begin{array}{rcl}\cos \,({k}_{x}({a}_{m}+{a}_{d})) & = & \cos \,({k}_{mx}{a}_{m})\,\cos \,({k}_{dx}{a}_{d})\\  &  & -\frac{1}{2}\,(\tfrac{{\varepsilon }_{m}{k}_{dx}}{{\varepsilon }_{d}{k}_{mx}}+\tfrac{{\varepsilon }_{d}{k}_{mx}}{{\varepsilon }_{m}{k}_{dx}})\,\sin \,({k}_{mx}{a}_{m})\,\sin \,({k}_{dx}{a}_{d})\end{array}$$

where ${k}_{mx}=\sqrt{{\varepsilon }_{m}{k}_{0}^{2}-{k}_{z}^{2}}$ and ${k}_{dx}=\sqrt{{\varepsilon }_{d}{k}_{0}^{2}-{k}_{z}^{2}}$. Generally, the rigorous band structure in Eq. (9) implies the relations among the wave vectors *k*_*x*_, *k*_*z*_, and the frequency *ω*, containing the information of both the IFC at a certain frequency *ω* and the dispersion relation at a certain wave vector *k*_*x*_ (especially at the Brillouin zone centre *k*_*x*_ = 0). Similar to the coupled-mode theory, the IFCs obtained from Eq. (9) for each metal-dielectric multilayer stack also possess two branches, in which the symmetric mode with wave vector *β*_*s*_ and the anti-symmetric mode with wave vector *β*_*a*_ can be defined at the Brillouin zone centre. Furthermore, the degeneracy of the symmetric mode and the anti-symmetric mode forms the Dirac points, leading to giant optical nonlocality in the metal-dielectric multilayer stacks. Figure 2(a) displays the dispersion relation between the normalized wave vector *k*_*z*_/*k*_*p*_ and the normalized frequency *ω*/*ω*_*p*_ at *k*_*x*_ = 0 for two metal-dielectric multilayer metamaterial stacks, where the plasma wave vector *k*_*p*_ = *ω*_*p*_/*c* is associated with the plasma frequency *ω*_*p*_ and the speed of light *c* in free space. The dispersion relations for the Au-SiO_2_ and Au-Al_2_O_3_ multilayer stacks are denoted by the blue curves and the red curves, respectively, including both the symmetric modes ${\beta }_{s}^{\mathrm{(1)}}$ and ${\beta }_{s}^{\mathrm{(2)}}$ and the anti-symmetric modes ${\beta }_{a}^{\mathrm{(1)}}$ and ${\beta }_{a}^{\mathrm{(2)}}$. Clearly, the degeneracy of the symmetric and anti-symmetric modes leads to the emergence of the Dirac points at the frequencies ${\omega }_{D}^{\mathrm{(1)}}$ and ${\omega }_{D}^{\mathrm{(2)}}$ for the Au-SiO_2_ and Au-Al_2_O_3_ multilayer stacks, respectively. According to the theory describing the Klein tunneling in the relativistic quantum mechanics, the frequency band for the Klein tunneling of optical wave to occur is from *ω*_*L*_ = 0.0788*ω*_*p*_ (173.098 THz) to *ω*_*H*_ = 0.120*ω*_*p*_ (262.559 THz) for the current two multilayer stacks, where *ω*_*L*_ is determined by the intersection of the dispersion curves for the symmetric mode ${\beta }_{s}^{\mathrm{(1)}}$ and the anti-symmetric mode ${\beta }_{a}^{\mathrm{(2)}}$, and *ω*_*H*_ is located at the intersection of the dispersion curves for the anti-symmetric mode ${\beta }_{a}^{\mathrm{(1)}}$ and the symmetric mode ${\beta }_{s}^{\mathrm{(2)}}$. Furthermore, as shown in Fig. 2(a) the frequency band of Klein tunneling is divided into three frequency ranges as I, II, and III, with respect to the locations of two Dirac points.

Figure 2(b) further shows the typical IFCs at the frequency of *ω* = 0.095*ω*_*p*_ (208.652 THz) in the frequency range II for the Au-SiO_2_ (solid blue curves) and Au-Al_2_O_3_ (solid red curves) multilayer stacks. For the Au-SiO_2_ multilayer stack, it is clear that the IFCs possess two different branches with the anti-symmetric mode ${\beta }_{a}^{\mathrm{(1)}}$ in the lower branch and the symmetric mode ${\beta }_{s}^{\mathrm{(1)}}$ in the upper branch at the Brillouin zone centre. The similar IFC profiles are also observed for the Au-Al_2_O_3_ multilayer stack, but with the ${\beta }_{a}^{\mathrm{(2)}}$ mode in the upper branch and the ${\beta }_{s}^{\mathrm{(2)}}$ mode in the lower branch. This is because the selected frequency *ω* = 0.095*ω*_*p*_ is located between the two Dirac point frequencies ${\omega }_{D}^{\mathrm{(1)}}$ and ${\omega }_{D}^{\mathrm{(2)}}$ for the two multilayer stacks and there is a band inversion across the Dirac points, which is coincident with the dispersion relations shown in Fig. 2(a). Besides, the IFCs obtained from the coupled-mode theory (dashed curves) are also plotted for comparison, indicating that the coupled-mode theory only works well around the Brillouin zone centre. Note that both the energy and momentum are conserved in the Klein tunneling for the relativistic electrons passing across a potential barrier. For the optical analogue of Klein tunneling working at a specific frequency *ω*, the wave vector *k*_*z*_ is conserved as the light waves refracted at the interface of two multilayer stacks. Therefore, as indicated in Fig. 2(b), the Klein tunneling zone in wave vector space can be directly obtained from the overlapped IFCs of two multilayer stacks sharing the same wave vector *k*_*z*_. Figure 2(b) marks the Klein tunneling zone for the wave vector *k*_*x*_ predicted by the transfer-matrix method with the overlap of the ${\beta }_{s}^{\mathrm{(1)}}$-branch and the ${\beta }_{s}^{\mathrm{(2)}}$-branch, as well by the coupled-mode theory with the overlap of the ${\beta }_{+}^{\mathrm{(1)}}$-branch and the ${\beta }_{-}^{\mathrm{(2)}}$-branch. It is worth emphasizing the Klein tunneling zones predicted from two approaches are different from each other, but they are coincident with each other at the Brillouin zone centre.

**Figure 3:**
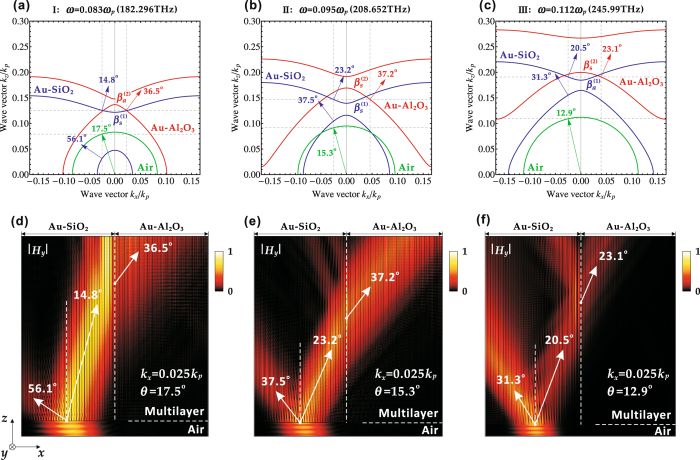
The IFC analysis based on the transfer-matrix method and the corresponding numerical simulation for the Klein tunneling at three different frequencies of (**a**,**d**) *ω* = 0.083*ω*_*p*_ (182.296 THz), (**b**,**e**) *ω* = 0.095*ω*_*p*_ (208.652 THz), and (**c**,**f**) *ω* = 0.112*ω*_*p*_ (245.99 THz) in the frequency range of I, II, and III, respectively. In the IFCs, the Poynting vectors representing the beam propagation directions are denoted by arrows. The absolute value of the magnetic component |*H*_*y*_| is plotted in the simulation, where the beam propagation direction of the TM polarized light beam is marked by arrows.

The IFCs obtained from the transfer-matrix method are used next to analyze the refraction processes at the interface of two multilayer stacks in the Klein tunneling of optical waves. For instance, Fig. 3 shows the IFCs at three different frequencies in the frequency range I (*ω* = 0.083*ω*_*p*_), II (*ω* = 0.095*ω*_*p*_), and III (*ω* = 0.112*ω*_*p*_) based on the transfer-matrix method. As depicted in Fig. 3(a), The IFCs at the frequency of *ω* = 0.083*ω*_*p*_ (182.296 THz) in the frequency range I indicate that an incident TM polarized light beam from air with 17.5° angle of incidence will excite two propagation modes within the Au-SiO_2_ multilayer stack, where the first mode belongs to the ${\beta }_{s}^{\mathrm{(1)}}$-branch with the beam propagation direction (the normal direction to the positive *z*-direction on the IFC) of 14.8°, and the second mode belongs to the ${\beta }_{a}^{\mathrm{(1)}}$-branch with the beam propagation direction of 56.1°. According to the momentum conservation condition for the Klein tunneling, the first mode can tunnel to the ${\beta }_{a}^{\mathrm{(2)}}$-branch in the Au-Al_2_O_3_ multilayer stack and excite the mode with the beam propagation direction of 36.5°. In order to further visualize the Klein tunneling of optical waves through two multilayer stacks, Fig. 3(d) displays the numerically simulated optical beam propagation and refraction processes inside the multilayer stacks. The simulation shows that an incident TM polarized Gaussian beam from air with 17.5° angle of incidence (*k*_*x*_ = 0.025 *k*_*p*_) is split into two propagating beams in the Au-SiO_2_ multilayer stack with one beam in the beam propagation direction of 14.8° tunneling into the Au-Al_2_O_3_ multilayer stack and the refraction angle is 36.5°. The similar optical mode propagation and tunneling processes for the Klein tunneling at the other two frequencies of *ω* = 0.095*ω*_*p*_ (208.652 THz) and *ω* = 0.112*ω*_*p*_ (245.99 THz) can also be found in Fig. 3(b,c,e,f), respectively. In addition, it is shown that the Klein tunneling of optical waves can take place between optical modes with different symmetries, depending on the operation frequency. Generally, in the frequency range I, the Klein tunneling arises from the mode tunneling between the symmetric ${\beta }_{s}^{\mathrm{(1)}}$-branch and the anti-symmetric ${\beta }_{a}^{\mathrm{(2)}}$-branch. In the frequency range II, the Klein tunneling occurs between the ${\beta }_{s}^{\mathrm{(1)}}$-branch and the ${\beta }_{s}^{\mathrm{(2)}}$-branch. And in the frequency range III, the Klein tunneling is between the ${\beta }_{a}^{\mathrm{(1)}}$-branch and the ${\beta }_{s}^{\mathrm{(2)}}$-branch.

**Figure 4:**
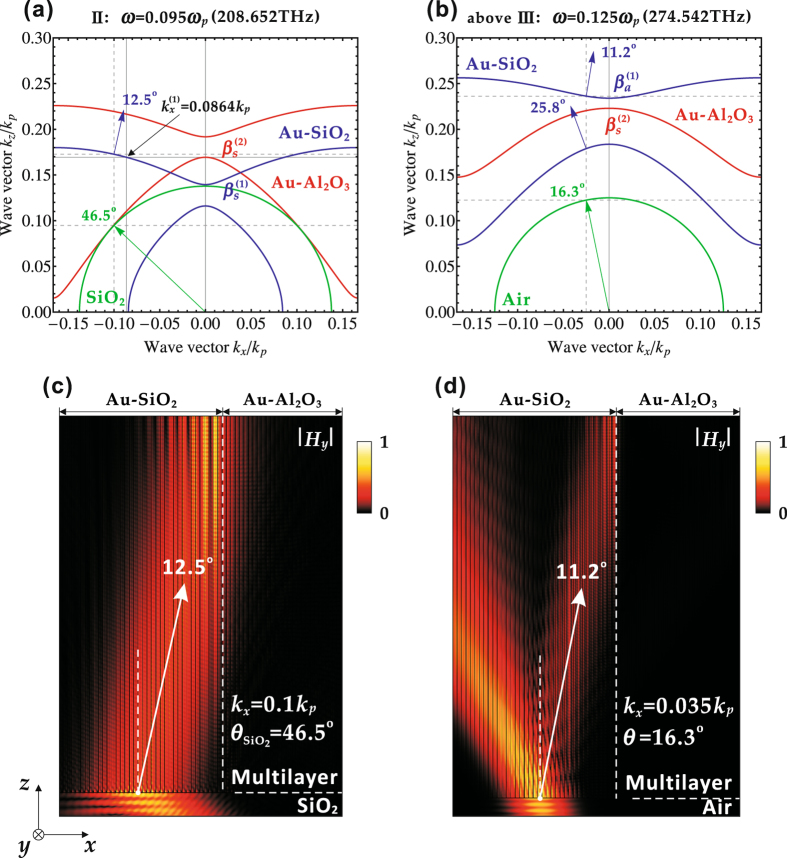
The IFC analysis and the corresponding numerical simulation for light propagation outside the Klein tunneling ranges, (**a**,**c**) at *ω* = 0.095*ω*_*p*_ (208.652 THz) in the frequency range II but with the wave vector out of the Klein tunneling zone, and (**b**,**d**) at *ω* = 0.125*ω*_*p*_ (274.542 THz) above the frequency range III.

For comparison, the propagation of the TM polarized light outside the Klein tunneling ranges in either the wave vector space or the frequency domain is also studied in Fig. 4. The first case displayed in Fig. 4(a,c) demonstrates the beam propagation at the frequency of *ω* = 0.095*ω*_*p*_ (208.652 THz) which is located in the frequency range II for the Klein tunneling, but with the wave vector *k*_*x*_ = 0.1 *k*_*p*_ out of the tunneling zone. The IFCs in Fig. 4(a) indicate that an incident TM polarized light from SiO_2_ with 46.5° angle of incidence (wave vector *k*_*x*_ = 0.1 *k*_*p*_) excites the mode in the beam propagation direction of 12.5° in the ${\beta }_{s}^{\mathrm{(1)}}$-branch of the Au-SiO_2_ multilayer stack. However, since no mode exists in the ${\beta }_{s}^{\mathrm{(2)}}$-branch of the Au-Al_2_O_3_ multilayer stack to match the wave vector *k*_*z*_, the Klein tunneling cannot occur. Correspondingly, Fig. 4(c) shows that the optical beam will not refract across the interface of two multilayer stacks based on the simulation, which is coincident with the IFC analysis. However, it is worth mentioning that there is still some optical energy evanescently couples cross the interface but it gets decayed quickly. The second case shown in Fig. 4(b,d) studies the beam propagation at the frequency of *ω* = 0.125*ω*_*p*_ (274.542 THz) which is above the frequency range III for the Klein tunneling. From the IFCs in Fig. 4(b), it is shown that an incident TM polarized light from air with 16.3° angle of incidence (wave vector *k*_*x*_ = 0.035 *k*_*p*_) excites two modes in the Au-SiO_2_ multilayer stack, with the beam propagation direction of 25.8° in the ${\beta }_{s}^{\mathrm{(1)}}$-branch and the beam propagation direction of 11.2° in the ${\beta }_{a}^{\mathrm{(1)}}$-branch. Since none of these two modes in the Au-SiO_2_ multilayer stack can match the mode in the ${\beta }_{s}^{\mathrm{(2)}}$-branch of the Au-Al_2_O_3_ multilayer stack with the equal wave vector *k*_*z*_, the Klein tunneling cannot occur. The simulation in Fig. 4(d) also clearly shows that the propagation mode with the direction of 11.2° in the Au-SiO_2_ multilayer stack is completely reflected back at the interface of two multilayer stacks.

### Transmission and reflection of Klein tunneling

Analogous to the Dirac equation describing the Klein tunneling for the relativistic electrons, the coupled-mode theory reveals the propagation and refraction processes of the TM polarized light waves tunneling across the interface of two multilayer stacks. Moreover, the coupled-mode theory can also be used to determine the transmission and reflection properties of the Klein tunneling of optical waves. With respect to the two multilayer stacks, the transmission coefficient *t* and the reflection coefficient *r* can be derived based on the coupled mode-theory as

10 $$t=\frac{-2{\bf{i}}{C}_{1}\,\sin \,({k}_{x}^{\mathrm{(1)}}a)}{{C}_{2}\,[1-\exp \,({\bf{i}}{k}_{x}^{\mathrm{(1)}}a)]\,\exp \,({\bf{i}}{k}_{x}^{\mathrm{(1)}}a)-{C}_{1}\,[1-\exp \,(-{\bf{i}}{k}_{x}^{\mathrm{(2)}}a)]\,\exp \,({\bf{i}}{k}_{x}^{\mathrm{(1)}}a)}$$

11 $$r=\frac{-2{\bf{i}}{C}_{2}\,\sin \,({k}_{x}^{\mathrm{(1)}}a)\,\exp \,(-{\bf{i}}{k}_{x}^{\mathrm{(1)}}a)}{{C}_{2}\,[1-\exp \,({\bf{i}}{k}_{x}^{\mathrm{(1)}}a)]\,\exp \,({\bf{i}}{k}_{x}^{\mathrm{(1)}}a)-{C}_{1}\,[1-\exp \,(-{\bf{i}}{k}_{x}^{\mathrm{(2)}}a)]\,\exp \,({\bf{i}}{k}_{x}^{\mathrm{(1)}}a)}-\exp \,(-{\bf{i}}2{k}_{x}^{\mathrm{(1)}}a)$$

with the parameters ${C}_{1}=({\beta }_{+}^{\mathrm{(1)}}+{\delta }^{\mathrm{(1)}}-{\beta }_{0}^{\mathrm{(1)}})/{\kappa }^{\mathrm{(1)}}$ and ${C}_{2}=({\beta }_{+}^{\mathrm{(2)}}+{\delta }^{\mathrm{(2)}}-{\beta }_{0}^{\mathrm{(2)}})/{\kappa }^{\mathrm{(2)}}$, the mode propagation constants ${\beta }_{0}^{\mathrm{(1)}}$ and ${\beta }_{0}^{\mathrm{(2)}}$, the propagation constant differences *δ* ^(1)^ and *δ* ^(2)^, and the coupling strengths *κ* ^(1)^ and *κ* ^(2)^, for the Au-SiO_2_ and Au-Al_2_O_3_ multilayer stacks, respectively [Appendix 2]. Furthermore, compared to the relativistic quantum mechanics theory for Klein tunneling, the intensity current for the TM polarized light in either of the two multilayer stacks can be defined as

12 $${J}_{n}={\bf{i}}\kappa ({A}_{2n}{A}_{2n+1}^{\ast }-{A}_{2n}^{\ast }{A}_{2n+1})$$

Therefore, the transmission and reflection of the Klein tunneling read as

13 $$T=|{J}_{{\rm{trans}}}/{J}_{{\rm{inc}}}|=|{C}_{2}/{C}_{1}|\,{|t|}^{2}$$

14 $$R=|{J}_{{\rm{ref}}}/{J}_{{\rm{inc}}}|={|r|}^{2}$$

**Figure 5:**
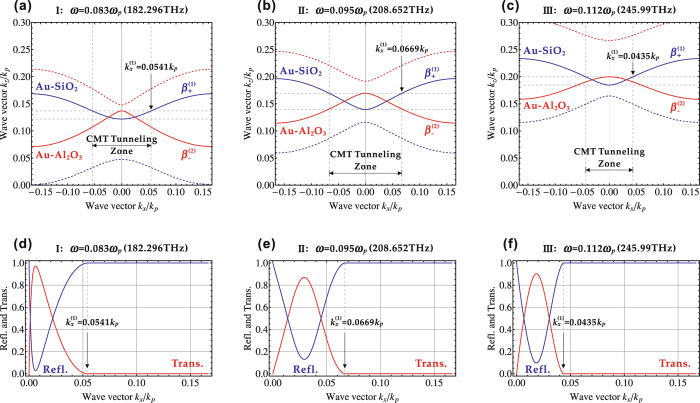
The IFCs based on the coupled-mode theory and the transmission and reflection spectra for the Klein tunneling at three different frequencies of (**a**,**d**) *ω* = 0.083*ω*_*p*_ (182.296 THz), (**b**,**e**) *ω* = 0.095*ω*_*p*_ (208.652 THz), and (**c**,**f**) *ω* = 0.112*ω*_*p*_ (245.99 THz) in the frequency range of I, II, and III, respectively. The IFCs related to the Klein tunneling are denoted by solid curves, while the others are denoted by dashed curves. The boundary of Klein tunneling zone in wave vector space is also marked.

According to Eqs (13) and (14), Fig. 5 plots the transmission and reflection spectra as a function of wave vector *k*_*x*_, together with the corresponding IFCs based on the couple-mode theory at three different frequencies considered in Fig. 3. The IFCs in Fig. 5(a–c) are obtained from the coupled-mode theory with both the *β*_+_ and *β*_−_ branches denoted for each multilayer stack, in which the two branches involved in the Klein tunneling are denoted by solid curves, while other branches are denoted by dashed curves. The boundaries of wave vector *k*_*x*_ for the Klein tunneling zone are also marked in the IFC figures. As shown in Fig. 5(d–f), the calculated transmission increases from zero to the maximum and then goes back to zero once the wave vector *k*_*x*_ is out of the Klein tunneling zone, where the incident light is totally reflected by the interface of two multilayer stacks. Correspondingly, the reflection just behaves in an opposite way. The transmission and reflection spectra match with the Klein tunneling zone predicted from the IFC figures. In addition, the results of transmission and reflection spectra also imply the relation of *T* + *R* = 1 under the lossless condition due to the energy conservation.

## Discussion

The Klein tunneling of optical waves across the interface of two lossless metal-dielectric multilayer metamaterial stacks is demonstrated through the theoretical analysis based on the coupled-mode theory and the transfer-matrix method, together with numerical simulation. The connection between the metal-dielectric multilayer metamaterial stack modeled with the coupled-mode theory and the relativistic fermions system described by the Dirac equation is revealed. The Klein tunneling of optical waves is clearly illustrated by the iso-frequency contour analysis and the numerical simulation to visualize the beam propagation and refraction processes across the interface of multilayer stacks, together with the calculated transmission and reflection spectra. The demonstration of the Klein tunneling of optical waves in metal-dielectric multilayer metamaterials near the Dirac points due to the coupling between surface plasmon polariton eigenmodes will create new frontiers in exploring the Dirac-cone physics associated with relativistic particle behaviors in condensed matter systems.

## Method

### Coupled-mode theory

In general, the coupled-mode theory can be expressed as

15 $$\frac{{\rm{d}}{\tilde{A}}_{\nu }}{{\rm{d}}z}=\sum _{\mu }\,{\bf{i}}{\kappa }_{\nu }^{\mu }{\tilde{A}}_{\mu }{{\bf{e}}}^{{\bf{i}}({k}_{\mu }-{k}_{\nu })z}$$

which describes the relation between the mode ${\tilde{A}}_{\nu }$ with wave vector *k*_*ν*_ along the *z*-direction and all other modes ${\tilde{A}}_{\mu }$ with wave vector *k*_*μ*_ along the same direction through the coupling strength ${\kappa }_{\nu }^{\mu }$. Mathematically, Eq. (15) can be rewritten as

16 $$-({\bf{i}}\frac{{\rm{d}}{A}_{\nu }}{{\rm{d}}z}+{k}_{\nu }{A}_{\nu })=\sum _{\mu }\,{\kappa }_{\nu }^{\mu }{A}_{\mu }$$

by introducing the inverse Fourier transform as ${\tilde{A}}_{\nu }={A}_{\nu }\,\exp \,(-{\bf{i}}{k}_{\nu }z)$ and ${\tilde{A}}_{\mu }={A}_{\mu }\,\exp \,(-{\bf{i}}{k}_{\mu }z)$. Regarding the coupled dielectric-metal-dielectric waveguide array depicted in Fig. 1(b), the coupled-mode theory in Eq. (16) reads

17 $$-\,({\bf{i}}\frac{{\rm{d}}}{{\rm{d}}z}+{k}_{2n}+{\kappa }_{2n}^{2n})\,{A}_{2n}={\kappa }_{2n}^{2n-1}{A}_{2n-1}+{\kappa }_{2n}^{2n+1}{A}_{2n+1}$$

18 $$-\,({\bf{i}}\frac{{\rm{d}}}{{\rm{d}}z}+{k}_{2n+1}+{\kappa }_{2n+1}^{2n+1})\,{A}_{2n+1}={\kappa }_{2n+1}^{2n}{A}_{2n}+{\kappa }_{2n+1}^{2n+2}{A}_{2n+2}$$

for the 2*n* mode and the 2*n* + 1 mode in the *n*th waveguide under the tight-binding approximation. With the modified wave vectors ${\beta }_{1}={k}_{2n}+{\kappa }_{2n}^{2n}$ and ${\beta }_{2}={k}_{2n+1}+{\kappa }_{2n+1}^{2n+1}$, and the coupling strength ${\kappa }_{{\rm{out}}}={\kappa }_{2n}^{2n-1}={\kappa }_{2n+1}^{2n+2}$ standing for the mode coupling between two adjacent waveguides and ${\kappa }_{{\rm{in}}}={\kappa }_{2n}^{2n+1}={\kappa }_{2n+1}^{2n}$ standing for the mode coupling within the same waveguides, Eqs (17) and (18) can be written as

19 $$-\,({\bf{i}}\frac{{\rm{d}}}{{\rm{d}}z}+{\beta }_{0}-\delta )\,{A}_{2n}={\kappa }_{{\rm{out}}}{A}_{2n-1}+{\kappa }_{{\rm{in}}}{A}_{2n+1}$$

20 $$-\,({\bf{i}}\frac{{\rm{d}}}{{\rm{d}}z}+{\beta }_{0}+\delta )\,{A}_{2n+1}={\kappa }_{{\rm{in}}}{A}_{2n}+{\kappa }_{{\rm{out}}}{A}_{2n+2}$$

with respect to the propagation constant *β*_0_ = (*β*_2_ + *β*_1_)/2 and the propagation constant difference *δ* = (*β*_2_ − *β*_1_)/2. By substituting the symbolic solutions in Eq. (3), the band structure (i.e., the eigenvalues) can be obtained as

21 $${\beta }_{\pm }({k}_{x},{\beta }_{0})={\beta }_{0}\pm {\kappa }_{{\rm{in}}}\sqrt{{(\delta /{\kappa }_{{\rm{in}}})}^{2}+1+{\sigma }^{2}+2\sigma \,\cos \,\phi }$$

with *σ* = *κ*_out_/*κ*_in_ based on Eqs (19) and (20). Note that the band structure in Eq. (21) must be coincident with the results of the results of the rigorous solution in Eq. (9) at the Brillouin zone centre, thus following the simple definitions that *β*_−_(0, *β*_0_) = *β*_1_ = min(*β*_*s*_, *β*_*a*_) and *β*_+_(0, *β*_0_) = *β*_2_ = max(*β*_*s*_, *β*_*a*_), there will be *σ* = *κ*_out_/*κ*_in_ = −1 and *κ*_in_ > 0 for Eq. (21). Simply speaking, since the coupled dielectric-metal-dielectric waveguide array is constructed by infinite identical single waveguide, the coupling strength *κ*_in_ and the coupling strength *κ*_out_ can be treated to have the same in magnitude but with a fixed phase shift. Therefore, by defining *κ*_in_ = −*κ*_out_ = *κ* > 0, the coupled-mode theory of Eqs (19) and (20) will be deduced into the simple format of Eqs (1) and (2), while the band structure of Eq. (21) will be deduced into the simple format of Eq. (4).

### Transmission and reflection coefficients

The transmission coefficient and the reflection coefficient are derived from the eigenvectors of Eqs (1) and (2) as

22 $$[\begin{array}{c}{A}_{2n}\\ {A}_{2n+1}\end{array}]=[\begin{array}{c}1-{{\bf{e}}}^{-{\bf{i}}\phi }\\ \sqrt{{(\delta /\kappa )}^{2}+2-2\,\cos \,\phi }+\delta /\kappa \end{array}]\,\exp \,({\bf{i}}{\beta }_{+}z+{\bf{i}}n\phi )$$

for *β*_+_-branch in Eq. (4), and

23 $$[\begin{array}{c}{A}_{2n}\\ {A}_{2n+1}\end{array}]=[\begin{array}{c}1-{{\bf{e}}}^{-{\bf{i}}\phi }\\ -\sqrt{{(\delta /\kappa )}^{2}+2-2\,\cos \,\phi }+\delta /\kappa \end{array}]\,\exp \,({\bf{i}}{\beta }_{-}z+{\bf{i}}n\phi )$$

for *β*_−_-branch in Eq. (4). Compared to the Klein tunneling in the relativistic quantum mechanics, the Klein tunneling should arise from the ${\beta }_{+}^{\mathrm{(1)}}$-branch in the Au-SiO_2_ multilayer stack to the ${\beta }_{-}^{\mathrm{(2)}}$-branch in the Au-Al_2_O_3_ multilayer stack. According to the conservation of the propagation constants, there will be

24 $$\begin{array}{c}{\beta }_{0}^{\mathrm{(1)}}+{\kappa }^{\mathrm{(1)}}\sqrt{{({\delta }^{\mathrm{(1)}}/{\kappa }^{\mathrm{(1)}})}^{2}+2-2\,\cos \,({k}_{x}^{\mathrm{(1)}}a)}\\ \quad ={\beta }_{0}^{\mathrm{(2)}}-{\kappa }^{\mathrm{(2)}}\sqrt{{({\delta }^{\mathrm{(2)}}/{\kappa }^{\mathrm{(2)}})}^{2}+2-2\,\cos \,({k}_{x}^{\mathrm{(2)}}a)}\end{array}$$

for the TM polarized light. Meanwhile, at the interface of the two different multilayer stacks, i.e., the interface between the −1th waveguide and the 0th waveguide (note that $n=\lfloor x/a\rfloor$), the amplitudes of the TM polarized light should also be conserved, which means

25 $$\begin{array}{c}[\begin{array}{c}1-\exp \,(-{\bf{i}}{k}_{x}^{\mathrm{(1)}}a)\\ ({\beta }_{+}^{\mathrm{(1)}}+{\delta }^{\mathrm{(1)}}-{\beta }_{0}^{\mathrm{(1)}})/{\kappa }^{\mathrm{(1)}}\end{array}]\,\exp \,(-{\bf{i}}{k}_{x}^{\mathrm{(1)}}a)+r\,[\begin{array}{c}1-\exp \,({\bf{i}}{k}_{x}^{\mathrm{(1)}}a)\\ ({\beta }_{+}^{\mathrm{(1)}}+{\delta }^{\mathrm{(1)}}-{\beta }_{0}^{\mathrm{(1)}})/{\kappa }^{\mathrm{(1)}}\end{array}]\,\exp \,({\bf{i}}{k}_{x}^{\mathrm{(1)}}a)\\ \quad =\,t[\begin{array}{c}1-\exp \,(-{\bf{i}}{k}_{x}^{\mathrm{(2)}}a)\\ ({\beta }_{+}^{\mathrm{(2)}}+{\delta }^{\mathrm{(2)}}-{\beta }_{0}^{\mathrm{(2)}})/{\kappa }^{\mathrm{(2)}}\end{array}]\end{array}$$

Based on Eqs (24) and (25), the transmission coefficient and the reflection coefficient can be obtained as in Eqs (10) and (11), respectively.
